# From diagnostics to prediction: development and validation of a multi-domain power-duration model

**DOI:** 10.1007/s00421-026-06142-8

**Published:** 2026-03-05

**Authors:** Patrick Wahl, Sanghyeon Ji

**Affiliations:** 1 Section Exercise Physiology, German Sport University Cologne, Am Sportpark Müngersdorf 6, 50933 Cologne, Germany; 2 The German Research Center for Elite Sport, Cologne, Germany

**Keywords:** Anaerobic power reserve, Performance diagnostics, Power prediction, Cycling, Power profiling

## Abstract

**Purpose:**

Current models of the power-duration relationship often focus on limited time domains. This study aims to develop and validate a Multi-Domain Power-Duration model (MuDo-PD) to predict power outputs across a wide range of exercise duration (up to 60 min) in cycling, using peak power output (PPO), maximal aerobic power (MAP), and power at lactate threshold 2 (P_LT2_).

**Methods:**

Thirty-three well-trained male cyclists (29.2 ± 9.7 yrs; V̇O₂max: 67.2 ± 5.1 mL·min⁻¹·kg⁻¹) performed lab tests to determine PPO (15-s sprint), MAP (ramp test), and P_LT2_, and completed time trials from 30 to 3600 s. Based on the resulting power-duration profiles and three anchor points (PPO, MAP, P_LT2_), individual exponential time decay constants (*k*) were calculated for short (1–300 s; Anaerobic Power Reserve, *k*_*AnPR*_) and long durations (300–3600 s; Aerobic Power Reserve, *k*_*AePR*_), forming the basis of the MuDo-PD model. Internal validation was performed within the modeling cohort by comparing the MuDo-PD to an established critical power approach (OmPD). External validation involved predicting the target power output during a time-to-exhaustion trial in an independent sample of 75 well-trained athletes.

**Results:**

Decay constants were *k*_*AnPR*_ = -0.023 ± 0.003 s^− 1^ and *k*_*AePR*_ = -0.0023 ± 0.0008 s^− 1^. The MuDo-PD model showed moderate to excellent agreement with actual power (ICC = 0.63–0.95; RSE = 29 ± 9 W), comparable to OmPD (ICC = 0.80–0.98, RSE = 19 ± 7 W). External validation confirmed excellent accuracy of MuDo-PD (ICC = 0.988; bias = 0.01 ± 17.8 W).

**Conclusion:**

The MuDo-PD model enables performance prediction across intensity domains up to 60 min using laboratory diagnostic parameters, offering a practical tool for performance assessment and training control.

**Supplementary Information:**

The online version contains supplementary material available at 10.1007/s00421-026-06142-8.

## Introduction

Over the last decade, a hyperbolic relationship between power output and exercise duration has been consistently demonstrated. This relationship helps explain the physiological limits of exercise tolerance and athletes’ performance across different intensity domains (Poole et al. 2016; Burnley and Jones 2018; Leo et al. 2022). To characterize this power-duration relationship in cycling, various models have been proposed, however, most models only cover specific intensity-domains of the power-duration relationship (Leo et al. 2022).

An exponential decline in power output has been observed for very short efforts (≤ 5 min), which is explained by the rapid depletion of the anaerobic energy supply as exercise duration increases. (Medbø et al. 1988; Medbø and Tabata 1993). In this context, the anaerobic power reserve (AnPR) model has emerged as a promising way of predicting short-duration power outputs in the domain of extreme exercise intensity (Weyand et al. 2006; Leo et al. 2022). The AnPR is defined as the difference between sprint peak power output (PPO; maximal neuromuscular and phosphagenic power output) and power output at V̇O₂max (the maximum aerobic power, MAP; maximal sustainable oxidative power (aerobic ceiling) that integrates both aerobic capacity and movement efficiency (Gastin 2001; Støren et al. 2013). The model assumes that the time‑dependent decline in high‑power performance follows the same relative pattern across athletes when scaled to their AnPR (Weyand et al. 2006). However, only two studies with rather small sample sizes have established an exponential constant (*k*) for cycling that describes the decrease in power output over time within AnPR. The study of Weyand et al. (2006) tested 7 moderately trained subjects (V̇O₂max: 49.2 ± 9.9 mL∙min^−1^∙kg^−1^) in a laboratory setting and established a single exponential constant of *k* = −0.026 s^−1^. Sanders and Heijboer (2019) used a field-testing approach with 12 professional cyclists (V̇O₂max 75 ± 6 mL∙min^−1^∙kg^−1^), establishing a slightly lower exponential constant of *k* = −0.0244 s^− 1^. Furthermore, in some studies (Sanders and Heijboer 2019; Weyand et al. 2006), the AnPR model’s predictive accuracy was not tested independently, because the exponential time constant (*k*) was taken from the same subjects whose performance the model later estimated. Therefore, a validation of the model in a larger and independent cohort of athletes is missing, as the studies primarily investigated the general validity of the model, rather than the specific degree of predictive accuracy.

Another approach similar to the AnPR model is the critical power (CP) model (Poole et al. 2016); however, the CP model mainly applies to describe the power-duration relationship during longer efforts (approximately 3–45 min). CP is typically determined using three to five tests lasting 2–15 min. This enables the calculation of CP using different methods such as weighted least square or geometric mean linear and nonlinear regression analysis. Although several CP‑based models exist, they often produce slightly different performance predictions, especially near the boundaries of the severe‑intensity domain (Muniz-Pumares et al. 2019; Leo et al. 2022). Depending on the model used, predictive accuracy varies for very short (extreme domain) and prolonged efforts (heavy domain) (Vandewalle et al. 1997; Dotan 2022). The most suitable model for predicting power outputs across a wide range of intensity domains is therefore still under discussion.

To improve whole‑range predictions (particularly to cover the moderate intensity domain), Puchowicz et al. (2020) recently developed the Omni Power Duration (OmPD) model using field-based maximal mean power (MMP) data across a wide range of durations (from 1 s to 4 h). The OmPD model combines an exponential decay function that describes the rapid depletion of the capacity of mechanical work above CP, called W´, i.e., extreme and severe intensity domains, and a log-linear fatigue term that accounts for the gradual performance decrements during prolonged exercise durations (i.e., heavy and moderate intensity domains). In a field-based validation using extensive MMP datasets from trained cyclists, the OmPD model demonstrated accurate fits across the full range of exercise durations, with particularly improved accuracy in both the extreme-intensity and moderate-intensity domains (Puchowicz et al. 2020). Despite its improved fit and broad applicability, the OmPD model does not directly account for underlying physiological mechanisms that may influence the shape of the power-duration relationship. Further, its accuracy depends heavily on the quality of field data (real all-out TT), which might limit its application for intraindividual longitudinal monitoring.

Beyond these power‑based models, key laboratory metrics such as the maximal lactate steady state (MLSS) or lactate threshold 2 (LT2) as an estimate of the MLSS, are generally regarded as alternative concepts and remain strong indicators of the upper limit of metabolic steady-state (Jones et al. 2019). Recent work suggests that differences between CP and MLSS largely stem from methodological factors (Caen et al. 2024). Power at LT2 (P_LT2_) calculated using Joyner’s model (1991) accurately estimates the power at MLSS (Keller et al. 2025) and shows high agreement with measured P_LT2_ (Fischer et al. 2025).

This raises the question of whether standard laboratory test outputs can be used to predict performance across a broad range of exercise durations besides these power-based models. Given the outcome parameters (e.g., P_LT2_, MAP, PPO) of typical laboratory performance tests (e.g., incremental tests, sprint test), the similarity of MLSS/P_LT2_ and CP, and a reported time-to-exhaustion (TTE) at MLSS/P_LT2_ of ~ 50–55 min (Faude et al. 2017; Baron et al. 2008), we propose extending the simple exponential approach used in the AnPR model to longer exercise durations (> 5–60 min). By incorporating MAP and P_LT2_, this approach forms a Multi‑Domain Power‑Duration model (MuDo‑PD).

To summarize, current models (AnPR, CP/OmPD) either lack physiological anchors or require extensive field data. Therefore, the MuDo-PD integrates anchors with exponential decay for a broader application.

Therefore, this study aimed to:

1. determine an exponent for an AnPR model using individual PPO and MAP, and power profiling data (ranging from 30 to 300 s) in a larger group of athletes.
2. develop an exponential aerobic power reserve model (AePR) using individual MAP, P_LT2_, and longer‑duration power‑profiling data (> 300–3600 s) in a large group of athletes.
3. validate whether the fusion of both models (AnPR and AePR) into the MuDo-PD accurately predicts power outputs across a wide range of exercise durations in both the same and an independent group of athletes.

## Methods

### Participants

A total of 98 well-trained athletes participated in the present studies. Descriptive values of anthropometry and performance data for each of the different cohorts are shown in Table 1. All study protocols were approved by the institutional review board (at the German Sport University Cologne, approval number 12/2015) and were performed in accordance with the Declaration of Helsinki. All participants were informed about the benefits and risks of the investigation prior to signing the institutionally approved informed consent document to participate in the study.

**Table 1:** Anthropometric and performance data of the athletes for the different cohorts

Used for	Study 1	Study 2	Study 3	Study 4	Study 5
	Establishing and interval validation of MuDo-PD model	External validation of MuDo-PD model			
Target time/intensity	TT 30–3600 s	TTE at estimated 3-min PO	TTE at MAP	TTE at 105% of P_LT2_	TTE at P_LT2_ & 105% of P_LT2_
Number of athletes Type of athletes	33 ♂ Cyclists	6 ♀, 16 ♂ Trained athletes	3 ♀, 12 ♂ Cyclists	3 ♀, 15 ♂ Cyclists/Triathletes	10 ♂ Cyclists/Triathletes
Age [years]	29.2 ± 9.65 (17–48)	24.4 ± 2.19 (21–29)	33.5 ± 9.36 (22–53)	28.9 ± 5.91 (19–38)	23.87 ± 5.28 (18–39)
Body Mass [kg]	75.0 ± 6.41 (62.8–86.9)	78.3 ± 13.1 (61.0–110.0)	77.1 ± 8.93 (58.1–88.0)	76.4 ± 11.0 (56.0–96.8)	78.8 ± 8.15 (67.7–90.4)
Height [cm]	183 ± 5.88 (173–197)	181 ± 7.28 (166–196)	178 ± 8.09 (161–187)	182 ± 8.59 (166–194)	185 ± 7.43 (174–195)
VO_2_max [mL∙min ^–1^ ]	5027 ± 417 (4074–6062)	4037 ± 870 (2574–6193)	4056 ± 514 (2654–4700)	4417 ± 710 (2643–5363)	4938 ± 428 (4248–5670)
[mL∙min^–1^∙kg^–1^]	67.2 ± 5.14 (58.1–81.0)	52.0 ± 10.1 (33.2–74.9)	52.6 ± 5.91 (43.9–62.2)	57.9 ± 6.96 (46.3–69.3)	63.0 ± 6.30 (55.9–74.3)
PPO [W]	1152 ± 160 (952–1716)	1026 ± 246 (597–1471)	981 ± 221 (594–1340)	1155 ± 288 (591–1596)	1155 ± 186 (867–1407)
MAP [W]	413 ± 29.9 (359–508)	347 ± 63.8 (249–466)	342 ± 45.1 (239–397)	359 ± 58.4 (233–445)	417 ± 28.5 (375–454)
P_LT2_ [W]	304 ± 29.0 (249–383)	-	237 ± 30.9 (187–272)	256 ± 41.7 (173–331)	302 ± 28.6 (260–354)
TTE [s]	-	190 ± 36.1 (130–253)	249 ± 67.4 (134–375)	1334 ± 583 (616–2304)	2064 ± 635 (1200–3600)

Data are presented as mean ± standard deviation (minimum-maximum)

*VO*_2_ max Maximal oxygen uptake, *PO* Power output, *PPO* Peak power output, *MAP* Maximal aerobic power, *P*_LT2_Power lactate threshold 2, *TT* Time trial, *TTE* Time to exhaustion

### Study 1: development of multi-domain power-duration model and its internal validation

In this study, 34 highly trained competitive cyclists completed 10 single-day testing sessions over a four-week period, with a minimum of 72 h rest between sessions. On the first and second testing days, each cyclist underwent a series of performance tests to determine PPO, MAP, and P_LT2_. Internal laboratory reliability values (Typical Error, TE) for these anchors – derived from cohorts with comparable performance characteristics – are as follows:

PPO: 39 W (3.5%),

MAP: 7.1 W (2.1%) and

P_LT2_: 6.4 W (2.5%).

During the remaining test sessions, each participant performed eight self-paced maximal time trials (TTs) ranging from 30 s to 3600 s, to create individual power-profiles (power-duration curves). The order of the TTs was randomized to minimize pacing-related learning effects. Based on the individual power-profiles, we determined an individual time decay constant (*k*) for power outputs ranging from 1 to 300 s (for AnPR model, *k*_*AnPR*_) and from 300 to 3600 s (for AePR model, *k*_*AePR*_) (see below). Utilizing the mean values of individual *k*_*AnPR*_ and *k*_*AePR*_, in conjunction with the fusion of the AnPR and AePR models (MuDo-PD), we predicted the power output for the different TT durations. We then assessed the agreement between the predicted and real power outputs to test the general validity of the models compared to the CP modelling approach (OmPD) (Puchowicz et al. 2020).

The time of day and ambient temperature (20–23 °C) remained consistent for each athlete throughout the test period. Participants were instructed to refrain from training for at least 24 h and to arrive rested, 2 h postprandial, and fully hydrated. All tests were conducted on an SRM ergometer (Schoberer Radmesstechnik, GmbH, Jülich, Germany), with individualized adjustments to the seat and handlebars, and each participant’s pedals mounted to the ergometer. Prior to each test, participants completed an intraindividual standardized warm-up on the cycle ergometer (i.e., at 2 W∙kg^− 1^ for 10 min followed by 5 min of passive rest).

### Performance tests-determination of model components

First, each participant performed a 15 s all-out sprint test in an isokinetic mode set to a cadence of 120 rpm (sampling frequency of 10 Hz), to determine peak power output (PPO; measured as a 1s peak power output) and mean power output. All participants were instructed to perform the test in a seated position on the ergometer with verbal encouragement provided throughout to help them achieve maximal power output.

After 20 min of recovery, participants performed an incremental ramp test, starting with an initial load of 160 W with an increment of 20 W·min^− 1^ until exhaustion to determine V̇O₂max (Metalyzer^®^3B; Cortex Biophysik GmbH, Leipzig, Germany), and MAP. Exhaustion was verified based on the following criteria (Midgley et al. 2007): respiratory exchange ratio ≥ 1.10, heart rate ≥ 95% of age-predicted maximum, blood lactate concentration ≥ 8 mmol·L^− 1^, and volitional exhaustion. The V̇O₂max and MAP were defined as the highest measured value of a 30 s and 60 s moving average during the ramp test, respectively.

On the second testing day, cyclists completed a step test starting at 160 W, increasing by 20 W every 3 min until volitional exhaustion, with capillary blood samples (20 µL) collected from the earlobe during the final 15 s of each step and analyzed immediately after the test (Biosen C-line, EKF-diagnostic GmbH, Barleben, Germany). Based on the linear regression model developed from the averaged VO_2_ data over the last 60 s of every stage (Metalyzer^®^3B; Cortex Biophysik GmbH, Leipzig, Germany), oxygen cost of cycling (Cc) was calculated at the power output representing 90% of LT2 (Fischer et al. 2025). Afterwards, P_LT2_ was determined as described previously using the following equation (Støren et al. 2013; Fischer et al. 2025; Keller et al. 2025):

$$P_{{LT2}} = LT2_{\% } ~ \cdot ~\frac{{VO_{2} \max }}{{Cc}}$$

This method has been shown to accurately estimate LT2 as well as MLSS (Fischer et al. 2025; Keller et al. 2025).

### Time trial tests–power profiling

During the remaining test sessions, the cyclists performed eight self-paced maximal TTs of varying durations (30, 60, 180, 300, 600, 1200, 2400, and 3600 s) in a randomized order. All TTs were performed on the SRM ergometer in the isokinetic mode with individually chosen cadences. During all TTs, constant feedback about the current power output, elapsed time, and HR was given. The athletes were verbally encouraged to reach their MMP. The power output was measured during the whole TT at 1 Hz. The mean power output across each respective TT was used as the representative power output (PO)-value for constructing the individual power profile.

### Modeling approaches–anaerobic and aerobic power reserve models

As introduced above, the AnPR model describes the power-duration relationship for short-duration exercises (≤ 300 s) by describing the exponential decline in power output between two anchor points – PPO and MAP. This decline is quantified by the exponential time decay constant *k*_*AnPR*_ (Weyand et al. 2006; Sanders and Heijboer, 2019):

$$PO\left( {t \le 300s} \right) = MAP + \left( {PPO - MAP} \right)\cdot~e^{{(k_{{AnPR}} \cdot t)}}$$

Based on the strong correlation (*r* = 0.814) and close absolute agreement (− 8 ± 38 W) between the MPO during 300-s TT and the MAP observed in this study – along with previous findings indicating a time to exhaustion (TTE) at MAP of approximately 253 ± 58 s (Moral-González et al. 2020) and an aerobic energy contribution during a ~ 300 s TT is > 85% (Gastin 2001) – we defined MAP as the lower boundary and 300 s as the lower time limit of the AnPR.

Building on the original AnPR concept, we developed an Aerobic Power Reserve (AePR) model to characterize the power-duration relationship across longer efforts (300–3600 s). Here, the anchor points are shifted: MAP serves as the upper limit, while P_LT2_ defines the lower boundary. Similar to the AnPR framework, we expected that an exponential time decay constant, *k*_*AePR*_, to characterize the progressive decline in power output over time between two anchor points, expressed as follows:

$$PO\left( {t> 300s} \right) = P_{{LT2}} + \left( {MAP - P_{{LT2}} } \right)\cdot~e^{{(k_{{AePR}} \cdot\left( {t - 300} \right))}}$$

To establish optimal time-decay constants for both the AnPR and AePR models, we used an iterative best-fit approach with nonlinear least-squares analysis. This means that the individual constants were determined by minimizing the sum of squared differences between the predicted and measured MPO during TTs across specific time domains: 1–300 s for AnPR and 300–3600 s for AePR. To ensure stable convergence of the nonlinear model, we employed a multi-start strategy, in which the decay constant (*k*_*AnPR*_ or *k*_*AePR*_) was estimated repeatedly across a pre-defined grid of initial values (−1.0 to 0 in 0.1 increments), retaining the solution with the lowest residual standard error (RSE) as the final fit. Based on these individually determined exponential decay constants, we established general time-decay constants by calculating the mean value across all participants, similar to previous studies (Weyand et al. 2006; Sanders and Heijboer 2019).

### Model fusion–multi-domain power-duration model (MuDo-PD)

To establish a unified framework for modeling the power-duration relationship across a wide range of exercise durations (1–3600 s), we developed the “MuDo-PD”, which seamlessly combines the AnPR and AePR approaches. This unified model incorporates exponential time decay functions within a continuous piecewise function – that is a model that applies different equations for different ranges of exercise duration. In this structure, each component governs a specific physiological domain: the AnPR component describes short-duration, anaerobically dominated efforts (≤ 300 s), while the AePR component models the decline in sustainable power during longer, predominantly aerobic efforts (> 300 s). The resulting piecewise model is defined as follows:

$$PO\left( t \right) = ~\left\{ {\begin{array}{*{20}c} {MAP + \left( {PPO - MAP} \right)\cdot~e^{{(k_{{AnPR}} \cdot t)}} ;~~if~t~ \le 300~s} \\ {P_{{LT2}} + \left( {MAP - P_{{LT2}} } \right)\cdot~e^{{(k_{{AePR}} \cdot\left( {t - 300} \right))}} ;~~~~if~t~> 300~s} \\ \end{array} } \right.$$

To illustrate how predicted power output is calculated, an example calculation is presented for a typical athlete (PPO = 1271 W, MAP = 508 W, P_LT2_ = 383 W) with the mean decay constants (*k*_*AnPR*_: −0.023, *k*_*AePR*_: −0.0023).

For a 180 s effort within the AnPR domain, the model yields:

$$PO_{{180}} = 508 + \left( {1271 - 508} \right)\cdot~e^{{ (- 0.023\cdot180)}} = 520~W$$

For a 1200 s effort within the AePR domain, the calculation is:

$$PO_{{1200}} = 383 + \left( {508 - 383} \right)\cdot~e^{{ (- 0.0023\cdot\left( {1200 - 300} \right))}} = 400~W$$

### Comparative modeling approach–Omni Power Duration (OmPD) model

As a comparison to our modelling approach (MuDo-PD), we have used the OmPD model, which has further developed the CP concept and proposed a way to characterize the power-duration relationship over a broad time spectrum within a unified mathematical structure (Puchowicz et al. 2020). The OmPD model is defined as a continuous piecewise function PO(t), with distinct formulations for exercise durations up to and beyond the maximum time to task failure at CP (CP_TTF_):

$$PO\left( t \right) = ~\left\{ {\begin{array}{*{20}c} {\frac{{W}}{t} \cdot \left( {1 - \exp ^{{ - t~ \cdot ~\frac{{PPO - CP}}{{W}}}} } \right) + CP;~~if~t~ \le CP_{{TTF}} } \\ {\frac{{W}}{t} \cdot \left( {1 - \exp ^{{ - t~ \cdot ~\frac{{PPO - CP}}{{W}}}} } \right) + CP - A \cdot \ln \left( {\frac{t}{{CP_{{TTF}} }}} \right);~~~if~t~> CP_{{TTF}} } \\ \end{array} } \right.$$

In this model, PO(t) is the predicted power output for a given exercise duration, PPO is the maximal instantaneous power, CP represents the critical power, Wʹ is the finite work capacity above CP, and A quantifies the log-linear fatigue describing the rate of decline in power with increasing exercise duration.

### Study 2: external validation of the multi-domain power-duration model

For external validation, the MuDo-PD model was applied using individual PPO, MAP, and P_LT2_ from studies 2–5, as well as the mean time-decay constants established in Study 1 – *k*_*AnPR*_ (−0.023 s^− 1^) and *k*_*AePR*_ (−0.0023 s^− 1^) – for predicting performance for efforts  ≤ 300 s and between 5 min and 60 min. The assessment of all individual performance landmarks – PPO, MAP, and P_LT2_ – in the external validation studies followed the same standardized procedures as described in Study 1.

In contrast to the approach used for the internal validation procedure in Study 1, where we used TT duration as the input to predict the corresponding MPO achieved during the effort, we used the participants’ TTE at given power outputs tailored to each individual as the input to the model to predict the individually prescribed target power outputs. These predicted values were then compared for absolute agreement with the actual target power outputs during the TTE trials in Studies 2–5 to evaluate the model’s external validity.

### Time to exhaustion

The TTE trial was performed after a standardized 10-minute warm-up at 2 W∙kg^− 1^. Participants performed the TTE trial at their preferred cadence. They did not receive any feedback regarding their elapsed time or the selected power output. Voluntary exhaustion was defined as the point at which participants’ cadence decreased by 10 rpm below their preferred cadence for 10 s, despite encouragement. The target intensity for the TTE trial in each study is detailed in Table 1.

### Statistical analysis

Statistical analysis was performed using R (R Core Team 2024; version 4.2.2). The normality of the data was checked by visual inspection of Q-Q plots. Non-linear models were fitted using *nls* function of the *stats* package. Considering that R^2^ may not adequately measure the goodness-of-fit for non-linear regression models (Spiess and Neumeyer 2010), we calculated the RSE for evaluating the goodness-of-fit of our models. This measure provides the mean distance of the regression line from raw observations, with a lower RSE indicating a better fit.

To characterize how parameter variability affects predictions within the MuDo-PD model, a sensitivity analysis was performed. Each model parameter (PPO, MAP, P_LT2_, and *k*_*AnPR*_ or *k*_*AePR*_) was independently varied by ± 5% while all remaining parameters were held constant, and the resulting deviations in predicted power output across the full duration range were then quantified.

To assess model performance – for both internal and external validation – a Bland-Altman analysis was performed with absolute and relative mean difference (i.e., fixed bias) and limits of agreement (i.e., random bias, ± 1.96 standard deviation). In addition, intra-class correlation coefficients (ICC) were calculated based on a single measure absolute agreement, a two-way random effect model, to examine the agreement between the real and predicted power outputs (*icc* package). According to Koo and Li (2016), the degree of agreement was interpreted as follows: < 0.50 = poor, 0.50–0.75 = moderate, 0.75–0.90 = good, and > 0.90 = excellent. Correlations between measured and model-predicted power outputs were determined using Pearson’s correlation coefficient *r* (*stats* package). These were interpreted as follows: < 0.30 = negligible, 0.30–0.50 = low, 0.50–0.70 = moderate, 0.70–0.90 = high, and > 0.90 = very high (Mukaka 2012). Model performance across the individual TT durations was further evaluated using different error metrics: mean absolute error (MAE), symmetric mean absolute percentage error (sMAPE), and root-mean-square error (RMSE). Differences between modelling approaches for MAE and RMSE were tested using the Diebold-Mariano test, based on absolute forecast errors for MAE and squared forecast errors for RMSE (*forecast* package). Differences in sMAPE between models were evaluated using paired t-tests (*stats* package).

Potential systematic bias and heteroscedasticity of the model prediction were assessed using residuals vs. predicted plots, which showed homogeneously distributed residuals across the full range of predicted power outputs without any evidence of systematic funneling or increasing variance (see supplementary Fig. S1).

For internal validation, statistical differences between actual and model-predicted power outputs were assessed using mixed-effects models (*lme4* package) treating *model* as a fixed effect (i.e., actual performance vs. MuDo-PD vs. OmPD) and *participants* as a random effect factor; significant main effects were followed by Bonferroni-corrected post-hoc comparisons (*emmeans* package). For external validation, statistical differences between actual and predicted power outputs were assessed using paired t-tests (*stats* package). An alpha level of 0.05 was interpreted as statistically significant.

### Sample size considerations

To evaluate whether the development cohort (*n* = 34) provided sufficient information for robust parameter estimation of the MuDo-PD model (i.e., *k*_*AnPR*_ and *k*_*AePR*_), we conducted a non-parametric 1000-iteration bootstrap analysis. In each iteration, athletes were resampled with replacement, and both decay constants were re-estimated using the same nonlinear fitting procedure applied in the primary model calibration. The bootstrap-derived estimates closely matched the empirically determined parameter values and exhibited very low sampling variability (*k*_*AnPR*_ = −0.023 ± 0.0004 s^− 1^ [95% CI, −0.024 – −0.022 s^− 1^]; *k*_*AePR*_ = −0.0023 ± 0.0001 s^− 1^ [95% CI, −0.0025 – −0.0021 s^− 1^]), indicating high parameter stability and supporting the adequacy of the development sample size.

For the external validation, an ICC-based power analysis (*ICC.Sample.Size* package) showed that detecting an agreement of ICC = 0.90 against a minimally acceptable ICC of 0.75 (k = 2, α = 0.05, power = 0.90) would require 44 participants. The external validation cohort (*n* = 75), which was retrospectively assembled from multiple independent studies, therefore exceeded the required sample size by a substantial margin, ensuring high statistical power.

## Results

Descriptive values of PPO, MAP, P_LT2_, and TTE are shown in Table 1 for each of the different studies/cohorts of athletes.

**Fig. 1:**
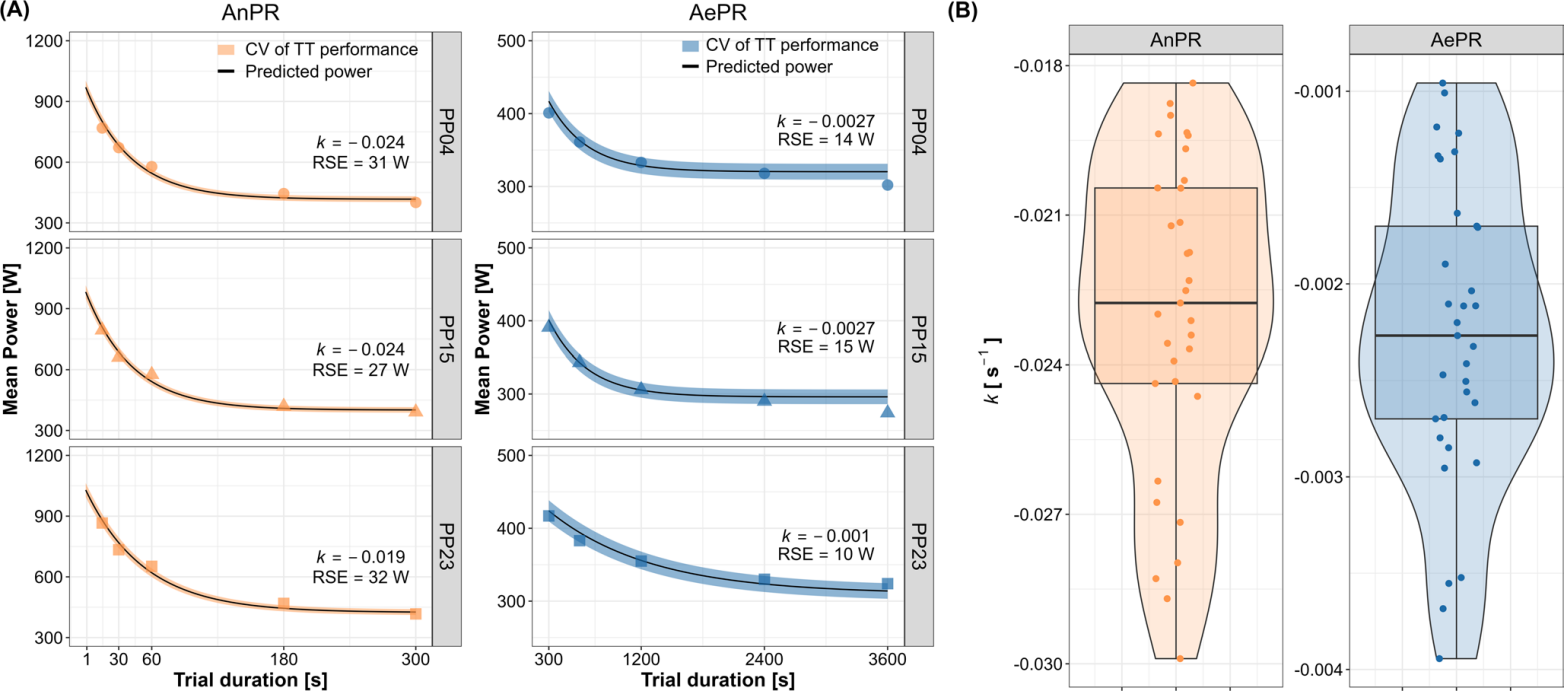
**A** Exemplary model fits for three representative athletes for the anaerobic power reserve (AnPR) and aerobic power reserve (AePR). The solid lines indicate the fitted power-duration relationship, and the shaded areas represent the CV (± 3.4%) for time trial performances (TT) from Currell & Jeukendrup (2008). **B** violin plots of the exponential constant (*k*), describing the decrement in power output over time for AnPR and AePR, respectively. RSE: residual standard error

Figure 1A shows exemplary fittings for three athletes for both the AnPR and the AePR models. The resulting individual exponential time-decay constants (Fig. 1B) were, on average, *k*_*AnPR*_ = −0.023 ± 0.003 s^− 1^ [95% CI, −0.024 – −0.022 s^− 1^] and *k*_*AePR*_ = −0.0023 ± 0.0008 s^− 1^ [95% CI, −0.0025 – −0.0020 s^− 1^]. The corresponding model fittings yielded a mean RSE of 32 ± 12 W [95% CI, 28–37 W] and 17 ± 8 W [95% CI, 14–20 W] for AnPR and AePR, respectively. Considering the day-to-day variability in TT performance (CV = 3.4%) reported in previous studies (Currell and Jeukendrup 2008), the predicted values are mainly within that corridor.

**Fig. 2:**
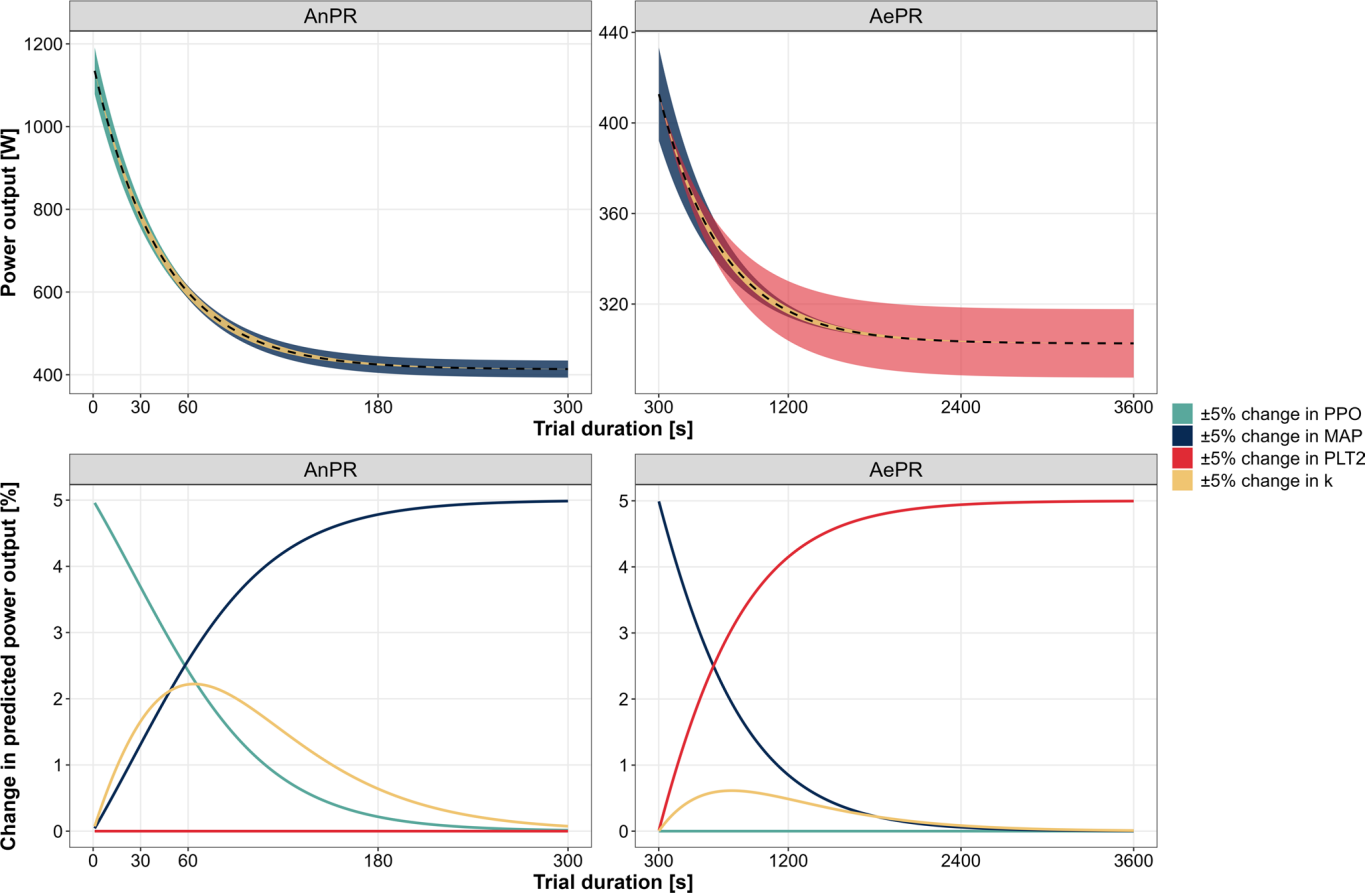
Sensitivity analysis of the multi-domain power-duration model. Top panels: Effect of ± 5% changes in peak power output (PPO), maximal aerobic power (MAP), the power at the second lactate threshold (P_LT2_), and the time-decay constants (k, i.e., *k*_*AnPR*_ or *k*_*AePR*_) on the predicted power-duration relationship in the anaerobic (AnPR, 1–300 s) and aerobic (AePR, 300–3600 s) domains. Dashed lines represent the predicted power output of the group mean; shaded areas indicate the change in predicted power output resulting from parameter variation. Bottom panels: Relative change in predicted power output (%) across trial durations for each ± 5% parameter variation

**Fig. 3:**
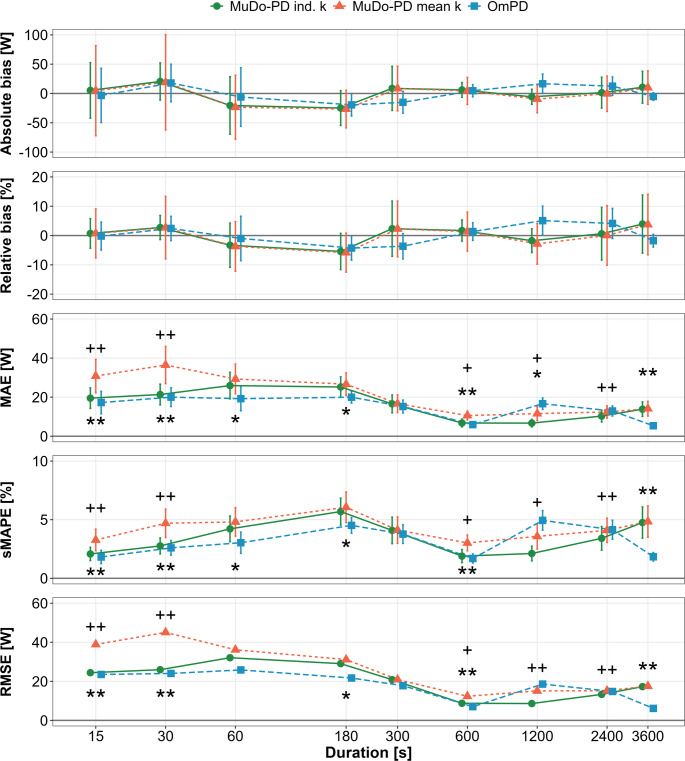
Model performance across the different time trial (TT) durations. Error metrics – absolute (difference between model-predicted – actual power output) and relative (% difference between model-predicted – actual power output) bias, mean absolute error (MAE), symmetric mean absolute percentage error (sMAPE), and root-mean-square error (RMSE) – are shown for the three modelling approaches: the multi-domain power–duration model (MuDo-PD) using individual time decay constants (MuDo-PD ind. *k*), MuDo-PD using mean time decay constants (MuDo-PD mean *k*), and the OmPD model. Metrics are presented across all TT durations (15–3600 s). Absolute and relative bias are displayed as mean ± limits of agreement (1.96 standard deviation), and MAE/sMAPE as mean ± 95% confidence interval. ^+^*p* < 0.05, ^++^*p* < 0.001 significant difference MuDo-PD ind. *k* vs. MuDo-PD mean *k*; ^*^p < 0.05, ^**^p < 0.001 significant difference MuDo-PD mean *k* vs. OmPD

To test whether exponential time-decay constants may covary with athlete characteristics, correlations between the individual decay constants (*k*_*AnPR*_ and *k*_*AePR*_) and athletes’ physiological and performance characteristics (e.g. V̇O_2_max, PPO, MAP, P_LT2_) were calculated. No meaningful correlations were found (−0.37 ≤ *r* ≤ 0.28, see supplementary Fig. S2). Furthermore, the sensitivity analysis (Fig. 2) showed that PPO exerted the strongest influence on the predicted power output (< 60 s), whereas MAP (60–600 s) and P_LT2_ (> 600 s) were the dominant determinants in longer durations. Variations in the time-decay constants (*k*_*AnPR*_ or *k*_*AePR*_) only had a moderate effect, primarily within the shorter time ranges of each domain. Although prediction errors were slightly smaller when individual constants were used (absolute bias: −0.13 ± 42.5 W vs. 1.54 ± 55.4 W; relative bias: −0.17 ± 8.92% vs. 0.18 ± 10.6%; MAE: 16.3 ± 1.63 W vs. 21.0 ± 2.17 W; sMAPE: 3.45 ± 0.33% vs. 4.28 ± 0.37%; RMSE: 21.6 W vs. 28.3 W), residual distributions were similar between the general (group mean *k*_*AnPR*_/*k*_*AePR*_) and individualized approaches (Fig. 3). Accordingly, the general time-decay constants were used for the MuDo-PD model in subsequent analyses.

**Fig. 4:**
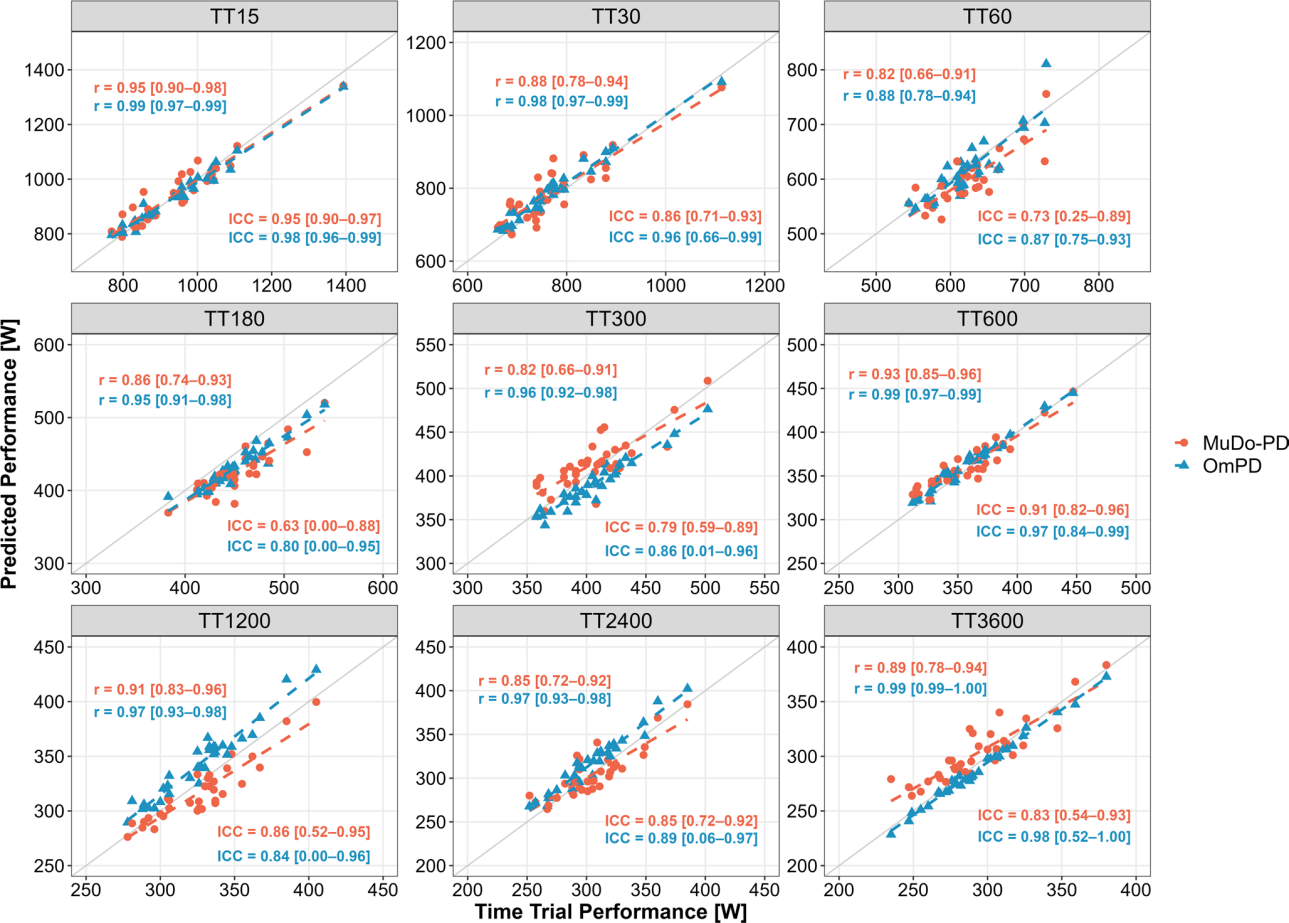
Internal model validation. Comparison between the predicted and actual power outputs for all time-trials (TT15 – TT3600) using the multi-domain power-duration model (MuDo-PD) and the Omni Power Duration model (OmPD). In each plot, Pearson’s correlation coefficients (*r* [95% confidence interval]) and intraclass correlation coefficients (ICC [95% confidence interval]) are reported. The solid grey line represents the line of identity, while the dashed lines showed the regression line for each model

Figure 4 shows the comparison of predicted and measured power outputs for all TTs using both the MuDo-PD and OmPD models. Both models showed high to very high correlations (*r* = 0.82–0.95 for the MuDo-PD model; *r* = 0.88–0.99 for the OmPD model) and moderate to excellent agreement with the actual performance data (ICC = 0.63–0.95 for the MuDo-PD model and ICC = 0.80–0.98 for the OmPD model). In the overall model performance analysis, the OmPD model demonstrated slightly higher predictive accuracy compared to the MuDo-PD model (absolute bias − 0.26 ± 37.3 W vs. 1.54 ± 55.4 W; relative bias − 0.24 ± 7.63% vs. 0.18 ± 10.6%; MAE 14.8 ± 3.94 W vs. 21.0 ± 2.17 W; sMAPE 3.16 ± 0.73% vs. 4.28 ± 0.37%; RMSE 19.0 W vs. 28.3 W). Across the individual TT durations, both models exhibited broadly comparable deviation patterns relative to the actual performances (Fig. 3). Detailed results for the actual vs. predicted TT performances, as well as the corresponding error metrics across different durations are presented in Table 2; Fig. 3.

**Table 2:** Mean power output (± standard deviation) during time trials (TT15 – TT3600), predicted values from the multi-domain power-duration model (MuDo-PD) and omni power duration (OmPD) models

	Actual power output [W]	Predicted power output		*p*-values from mixed effect model
		MuDo-PD[W]	OmPD [W]	
TT15	932 ± 125	937 ± 115	928 ± 111	0.269
TT30	765 ± 88	784 ± 83	783 ± 84	< 0.001 (MuDo-PD ≈ OmPD > Actual PO)
TT60	623 ± 45	600 ± 47	617 ± 54	< 0.001 (MuDo-PD > Actual PO ≈ OmPD)
TT180	451 ± 32	425 ± 30	432 ± 30	0.026 (Actual PO > OmPD > MuDo-PD)
TT300	405 ± 33	414 ± 30	390 ± 29	< 0.001 (MuDo-PD > Actual PO > OmPD)
TT600	356 ± 31	359 ± 28	359 ± 31	0.026 (MuDo-PD ≈ OmPD > Actual PO)
TT1200	327 ± 29	317 ± 27	343 ± 32	< 0.001 (OmPD > Actual PO > MuDo-PD)
TT2400	304 ± 29	304 ± 27	317 ± 31	< 0.001 (OmPD > MuDo-PD ≈ Actual PO)
TT3600	293 ± 32	303 ± 27	287 ± 31	< 0.001 (MuDo-PD > Actual PO > MuDo-PD)

Significant differences between actual and predicted values based on post-hoc comparisons are indicated as > (*p* < 0.05) or ≈ (*p* > 0.05)

**Fig. 5:**
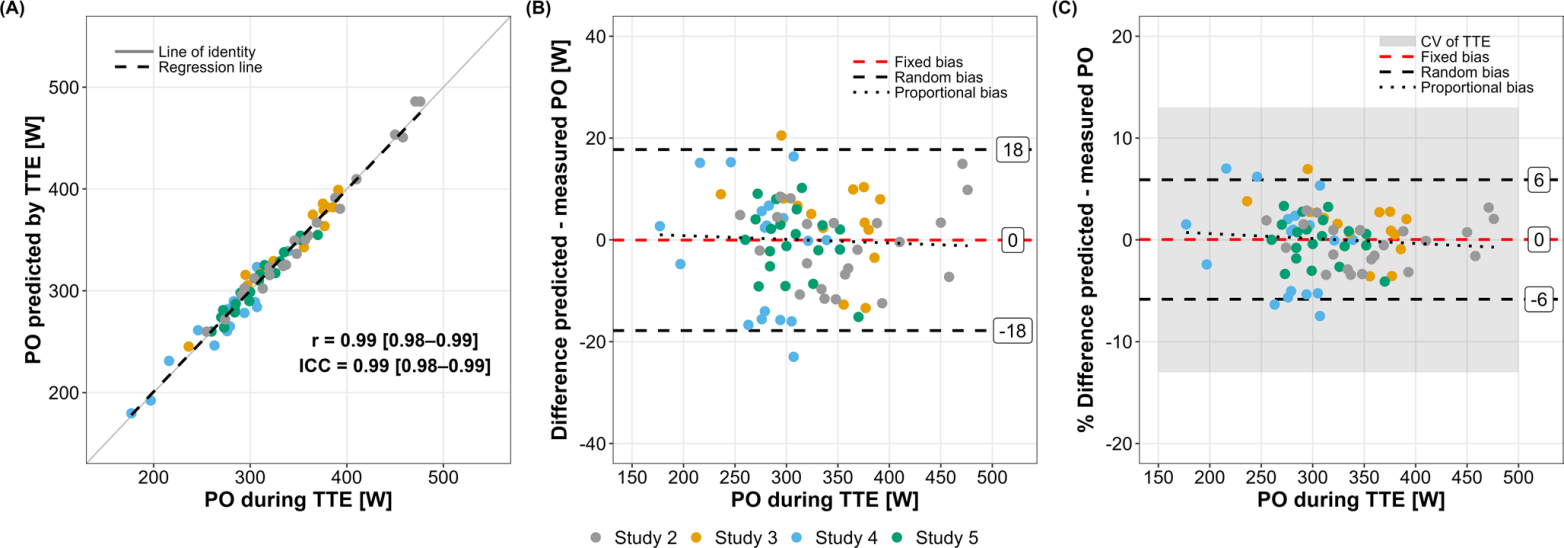
External model validation. **A** Scatterplot comparing the individually prescribed constant power output (PO) during the time-to-exhaustion (TTE) trial with the PO predicted by the MuDo-PD model. The solid grey line represents the line of identity, and the black dashed line shows the fitted linear regression. **B** and **C** Bland-Altman plots illustrating the absolute and percentage differences between the actual and model-predicted PO. The red dashed line denotes the mean difference (fixed bias), the black dashed lines indicate the upper and lower limits of agreement (mean ± 1.96 standard deviation; random bias), and the black dotted line represents the fitted linear regression (proportional bias). The shaded area reflects the mean coefficient of variation (CV) for TTE performance reported in previous studies (Inoue et al. 2023; Jeukendrup et al. 1996; Laursen et al. 2003)

In a second step, the predicted power outputs using the MuDo-PD model was then compared with the actual target power outputs during TTE from independent validation studies (Fig. 5). Predicted and actual target power outputs showed an almost perfect correlation and agreement (*r* = 0.99 and ICC = 0.99), with no significant difference between them (*p* = 0.15). The Bland-Altman analysis (Fig. 5B and C) revealed a small mean bias and narrow limits of agreement (absolute bias: 0 ± 18 W; relative bias: 0 ± 6%), with all data points falling within the expected day-to-day variability for TTE performance (mean CV = 13%) (Faude et al. 2017; Inoue et al. 2023; Jeukendrup et al. 1996; Laursen et al. 2003). The absolute and relative bias for each single study were as follows: Study 2: 0.88 ± 15.6 W and 0.27 ± 4.33%; Study 3: −3.95 ± 17.1 W and − 1.33 ± 5.23%; Study 4: 1.93 ± 24.0 W and 0.54 ± 8.79%; Study 5: 0.30 ± 13.0 W and 0.05 ± 4.19%.

## Discussion

This study aimed to establish and validate an exponent-based model (MuDo‑PD) for predicting cycling performance across a wide range of exercise durations/intensity domains. Specifically, we tested whether laboratory-derived metrics − peak power output (PPO), maximal aerobic power (MAP), and power at lactate threshold (P_LT2_) − together with two model exponents (*k*_*AnPR*_ and *k*_*AePR*_), could accurately predict power outputs for short (≤ 300 s) and longer (5–60 min) efforts. We also compared our results with the OmPD model (Puchowicz et al. 2020) and validated our approach in independent groups of athletes.

Firstly, the mean constant of the AnPR model (*k*_*AnPR*_ = −0.023 s^− 1^; Fig. 1), derived from a larger group of athletes, is very similar to the values reported by Sanders et al. (2019) (*k* = −0.0244 s^− 1^), and Weyand et al. (2006) (*k* = −0.026 s^− 1^). The predictive ability of the AnPR model using our constant *k*_*AnPR*_ is therefore comparable to that of previous studies. Previous studies using different constants reported prediction errors of 34 W (4.3%) to 53 W (6.6%) for short efforts (5–350 s) (Sanders et al. 2017; Sanders and Heijboer 2019; Weyand et al. 2006). Our model showed a MAE of 28 W and sMAPE of 4.6% for efforts lasting 15–300 s, which falls well within this established error range.

However, the exponent constant *k*_*AnPR*_ may vary depending on which measure is used as the lower bound of the AnPR. Sanders and Heijboer (2019) showed that modifying the lower bound of the AnPR (peak power output of an incremental test vs. 3 min all-out MPO) had the biggest impact on improving the predictive ability of their model. Therefore, while they concluded that the AnPR model’s applicability is limited for predicting power outputs beyond 180 s, our data do not support this claim. Using the MAP (the peak power output during our ramp test) and 300 s as the lower time boundary of the AnPR model, we were still able to confirm its high predictive accuracy. Consistent with previous studies indicating a TTE at MAP of approximately 254 s (Moral-González et al. 2020), the present data revealed high correlation (*r* = 0.814) and even absolute agreement (−8 ± 38 W) between the MPO of the 300 s TT and the MAP determined in our ramp incremental test. This high correlation might partly explain the high predictive ability of our AnPR model (Fig. 4).

To model performance over longer durations (300–3600 s), we introduced the AePR model using MAP and P_LT2_ as anchors. The mean exponential time decay constant (*k*_*AePR*_) = −0.0023; Fig. 1) showed a high level of goodness-of-fit (RSE = 17 ± 8 W), indicating that an exponential function can effectively describe the power‑duration relationship between MAP and P_LT2_. However, it should be noted that the variability of test protocols and P_LT2_-determination methods (Jamnick et al. 2020) might have an impact on the external validity of the AePR model.

It is worth noting that using individual *k* values consistently resulted in lower errors; however, the overall error patterns of individual versus mean *k* approaches remained similar (Fig. 3). Furthermore, the differences in MAE between the mean *k* and individual *k* approaches are relatively small (16.3 W vs. 21.0 W). Therefore, the mean *k* approach, together with PPO, MAP, and P_LT2_, offers a practical alternative that avoids extensive testing (multiple all-out time trials) and still delivers acceptable precision. The greater deviations for the very short TT (< 30 s) might be “negligible”, as these can be easily tested with low burden. Moreover, in our sample we did not observe meaningful associations between individual decay constants (*k*_*AnPR*_, *k*_*AePR*_) and athletes’ physiological or performance characteristics (V̇O_2_max, cycling economy, PPO, MAP, P_LT2_, see supplementary Fig. S2), indicating that *k* did not systematically covary with these parameters in our cohort. Taken together, although individualized *k* offers higher accuracy, mean *k* provides a pragmatic alternative with only modest loss of precision, thereby facilitating its use in diagnostic and training contexts.

Combining both domains, the MuDo‑PD model provides a unified prediction across short and long durations. Despite slightly lower correlations and ICCs for the MuDo-PD model (mean *r* = 0.88, mean ICC = 0.82) compared to the OmPD model (mean *r* = 0.96, mean ICC = 0.91) (Fig. 4), when applied to the data of Study 1, the present study shows that landmarks from laboratory tests such as PPO, MAP, and P_LT2_ can be used to accurately predict performance for up to 60 min (MAP: 21 W, sMAPE: 4.3%). The superior accuracy of the OmPD model is expected, given that it relies on MMP data from an athlete’s training history. From a practical standpoint, this data-driven approach offers flexible and broad applicability in performance modelling, particularly when comprehensive training or competition data are available. However, this dependence on high-quality, broad‑range (all-out) field data might also limit its use in many applied settings, such as intraindividual longitudinal monitoring, where data quality may vary over time.

The MuDo‑PD model requires only standard laboratory test results (PPO, MAP, P_LT2_) and fixed decay constants. This reduces testing burden and allows consistent application even when comprehensive field data are unavailable. Its dual‑anchor structure might also offer advantages for training prescription, as previous studies have shown that intensity zones defined by two anchors can yield more consistent physiological responses than zones based on a single maximal value (Wang and Zhao 2023, Luo et al. 2024, Lansley et al. 2011).

The model’s accuracy in previous studies (Weyand et al. 2006; Sanders and Heijboer 2019; Puchowicz et al. 2020) was not tested independently, as the value of the exponential time constant *k* was derived from the same subjects whose performance was subsequently estimated. In the present study, the validation in independent cohorts showed excellent agreement (ICC = 0.99) and very narrow limits of agreement (mean difference 0 ± 18 W; 0 ± 6%) (Fig. 5). This accuracy exceeded that of the OmPD model applied to Study 1 and the original study (standard error of the estimate of 3–9% MMP (1–10000 s) vs. model-predicted power output (Puchowicz et al. 2020), and fell within the typical variability (CV) of TTE performance (Faude et al. 2017; Inoue et al. 2023; Jeukendrup et al. 1996; Laursen et al. 2003). Because the validation sample lacked sufficient maximal mean power data, the OmPD model could not be applied.

### Limitations

Although this is the first study to perform an external validation of the model, certain limitations should be acknowledged. The validation TTEs only included a restricted duration range (130–300 s for AnPR; 616–3600 s for AePR). Therefore, further investigation is needed to assess the model’s accuracy over shorter durations within each respective domain. While the sample included a small number of female athletes, it was not sufficiently powered to examine potential sex-specific differences. Future studies should investigate the applicability and validity of the model in female athletes and more diverse populations. Further, the current MuDo-PD model has only been evaluated in cycling, and its transferability to other endurance sports like rowing or running remains unclear. Nevertheless, its conceptual structure suggests potential applicability beyond cycling, which should be investigated in future studies.

## Conclusion

In conclusion, the MuDo-PD model offers a practical and accessible tool for performance prediction and training control, as it relies solely on standard laboratory metrics (PPO, MAP, P_LT2_) and fixed decay constants. Beyond accurate modelling, the resulting individualized power-duration curves may support more precise training prescriptions by linking intensity and duration targets. Furthermore, the model’s dual-anchor structure aligns with evidence showing that training zones based on two reference points evoke more consistent physiological responses than those derived from a single maximal value. Together, these features highlight the MuDo-PD model’s potential to enhance individualized and evidence-based training planning.

## Supplementary Information

Below is the link to the electronic supplementary material.

Supplementary Material 1. Figure S1. Residuals vs. predicted plots for the multi-domain power-duration model using individual time-decay constants (MuDo-PD ind. k), using mean time-decay constants (MuDo-PD mean k), and Omni Power Duration model (OmPD). Residuals are plotted against predicted power output for all timetrial durations (15 – 3600 s). Solid lines with shaded bands represent loess-smoothed trends with 95% confidence intervals. Residuals were broadly homogeneously distributed across predicted power outputs, with no indication of systematic funneling or increasing variance.

Supplementary Material 2. Figure S2. Correlation (Pearson correlation coefficients) matrix of physiological characteristics, performance markers, and model parameters. abs. VO : absolute maximal oxygen uptake [mL⸱min ]; rel. VO : relative maximal oxygen uptake [mL⸱min ⸱kg ]; bLapeak_Sprint: peak blood lactate concentration following a 15-s maximal sprint test [mmol⸱L ]; PPO: maximal sprint peak power output [W]; MAP: maximum aerobic power [W]; PLT2: power output at the second lactate threshold; abs. OCc: absolute oxygen cost of cycling [mL⸱W ]; rel. OCc: relative oxygen cost of cycling [mL⸱W ⸱kg ]; AnPR: anaerobic power reserve (PPO - MAP) [W]; AePR: aerobic power reserve (MAP - PLT2) [W]; kAnPR: individual exponential time-decay constant within AnPR domain [s ]; kAePR: individual exponential time-decay constant within AePR domain [s ]. No meaningful correlations were observed between the time-decay constants and the physiological or performance variables.

## Data Availability

The datasets generated during and/or analyzed during the current study are available from the corresponding author on reasonable request.
