# Supplementary figures and images for: From diagnostics to prediction: development and validation of a multi-domain power-duration model
Source: Eur J Appl Physiol. 2026 Mar 5;126(6):3533–45. doi: 10.1007/s00421-026-06142-8 (PMC13287245; doi:10.1007/s00421-026-06142-8)

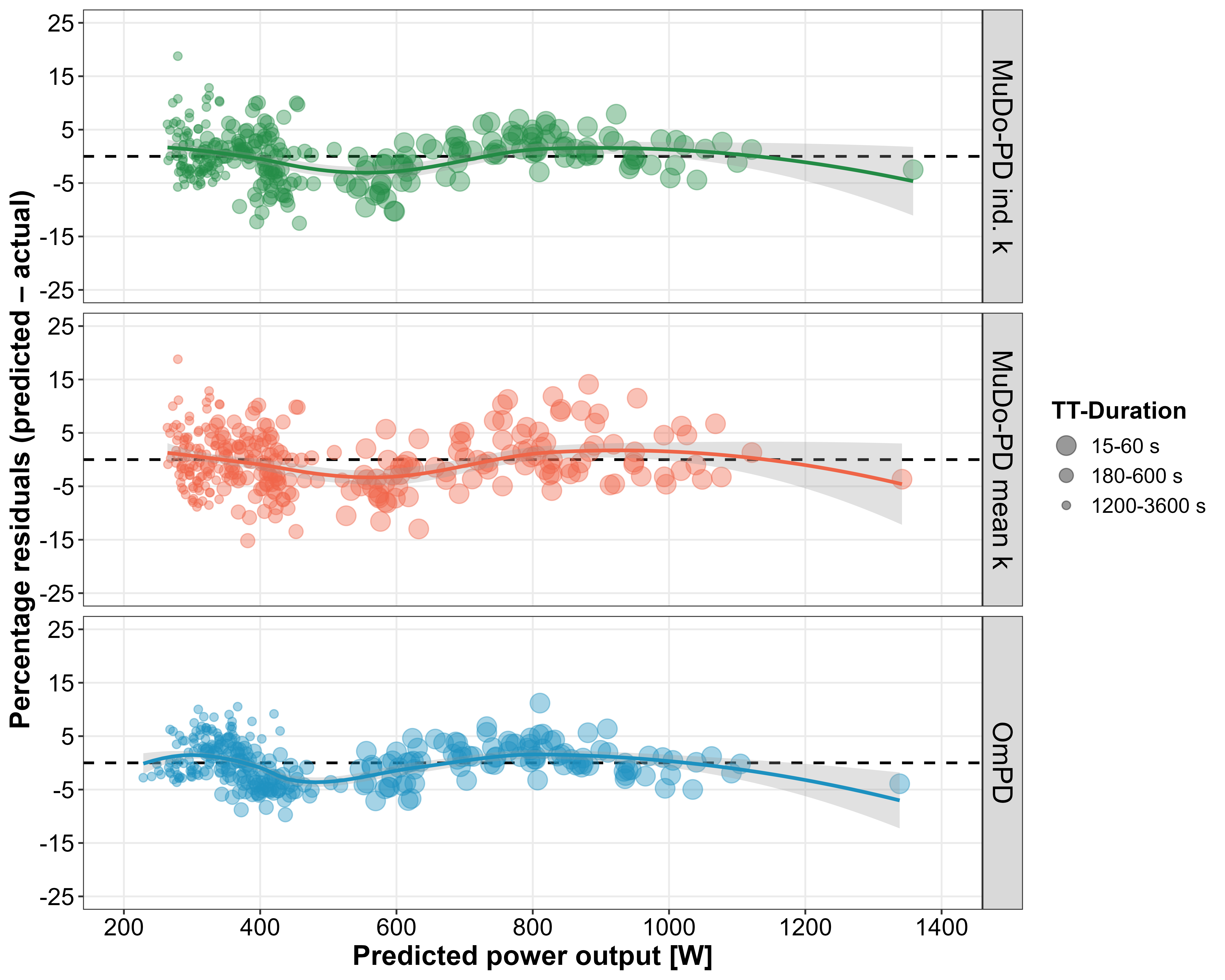

Supplement: Supplementary file 1 — Supplementary Material 1. Figure S1. Residuals vs. predicted plots for the multi-domain power-duration model using individual time-decay constants (MuDo-PD ind. k), using mean time-decay constants (MuDo-PD mean k), and Omni Power Duration model (OmPD). Residuals are plotted against predicted power output for all timetrial durations (15 – 3600 s). Solid lines with shaded bands represent loess-smoothed trends with 95% confidence intervals. Residuals were broadly homogeneously distributed across predicted power outputs, with no indication of systematic funneling or increasing variance. [file 421_2026_6142_MOESM1_ESM.png]

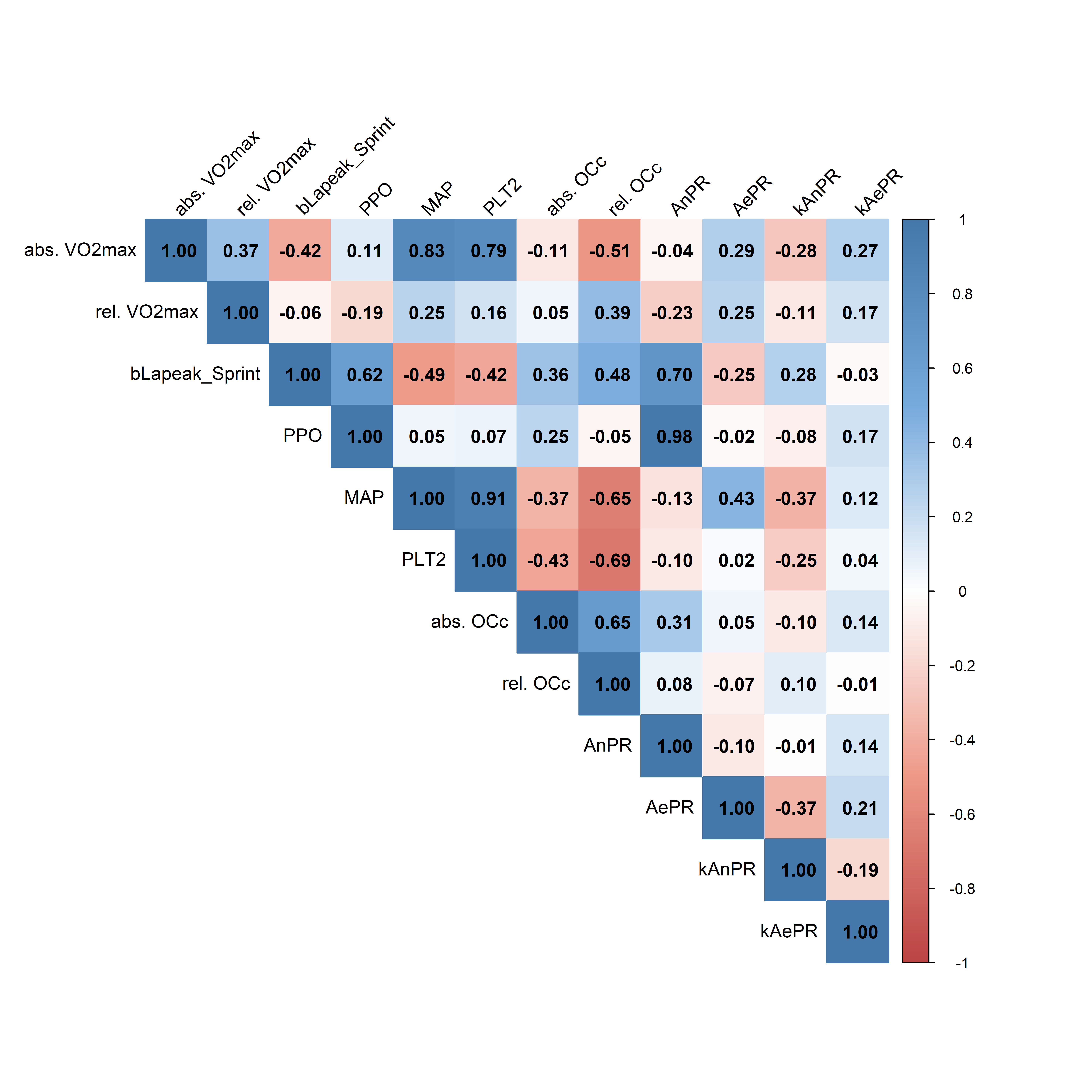

Supplement: Supplementary file 2 — Supplementary Material 2. Figure S2. Correlation (Pearson correlation coefficients) matrix of physiological characteristics, performance markers, and model parameters. abs. VO : absolute maximal oxygen uptake [mL⸱min ]; rel. VO : relative maximal oxygen uptake [mL⸱min ⸱kg ]; bLapeak_Sprint: peak blood lactate concentration following a 15-s maximal sprint test [mmol⸱L ]; PPO: maximal sprint peak power output [W]; MAP: maximum aerobic power [W]; PLT2: power output at the second lactate threshold; abs. OCc: absolute oxygen cost of cycling [mL⸱W ]; rel. OCc: relative oxygen cost of cycling [mL⸱W ⸱kg ]; AnPR: anaerobic power reserve (PPO - MAP) [W]; AePR: aerobic power reserve (MAP - PLT2) [W]; kAnPR: individual exponential time-decay constant within AnPR domain [s ]; kAePR: individual exponential time-decay constant within AePR domain [s ]. No meaningful correlations were observed between the time-decay constants and the physiological or performance variables. [file 421_2026_6142_MOESM2_ESM.png]
